# CHANGES OVER TIME IN DAYTIME SLEEPINESS AMONG OLDER ADULTS RECEIVING LONG-TERM SERVICES AND SUPPORTS

**DOI:** 10.1093/geroni/igz038.176

**Published:** 2019-11-08

**Authors:** Darina V Petrovsky, Karen B Hirschman, Glenna Brewster, Alexandra L Hanlon, Liming Huang, Miranda Varrasse McPhillips, Nancy A Hodgson, Mary D Naylor

**Affiliations:** 1 University of Pennsylvania, Philadelphia, Pennsylvania, United States; 2 Nell Hodgson Woodruff School of Nursing Emory University, Atlanta, Georgia, United States; 3 Virginia Polytechnic Institute and State University, Roanoke, Virginia, United States

## Abstract

The purpose of this study was to examine the predictors of excessive daytime sleepiness (EDS) over the first two years of long-term services and supports (LTSS) for 470 older adults in assisted living communities (ALCs), nursing homes (NHs), or their homes. Mixed effects linear regression modeling using a backward elimination process was used to build a final multivariable model. In the final model, being female (p<0.001) and fewer functional deficits (p<0.001) at baseline were associated with decreases in EDS, while higher baseline measures of BMI (p=0.004) and number of symptoms (p<0.001) were associated with higher EDS. Compared to older adults living in the community and receiving LTSS, those in NHs and ALCs had higher EDS (p<0.001). Greater feelings of belonging and depressive symptoms at baseline were associated with slower rates of increase in EDS over time (both p<0.001). Modifiable predictors of EDS and clinical implications will be discussed.

